# Adenoma development in familial adenomatous polyposis and MUTYH‐associated polyposis: somatic landscape and driver genes

**DOI:** 10.1002/path.4643

**Published:** 2015-11-02

**Authors:** Mamunur Rashid, Andrej Fischer, Cathy H Wilson, Jessamy Tiffen, Alistair G Rust, Philip Stevens, Shelley Idziaszczyk, Julie Maynard, Geraint T Williams, Ville Mustonen, Julian R Sampson, David J Adams

**Affiliations:** ^1^ Experimental Cancer GeneticsWellcome Trust Sanger InstituteHinxtonCambridgeUK; ^2^ Population Genomics of AdaptationWellcome Trust Sanger InstituteHinxtonCambridgeUK; ^3^ The Cancer Genome ProjectWellcome Trust Sanger InstituteHinxtonCambridgeUK; ^4^ Institute of Medical GeneticsCardiff University School of MedicineUK; ^5^ Institute of Cancer and GeneticsCardiff University School of MedicineUK

**Keywords:** colorectal neoplasms, adenoma development, somatic landscape, driver genes, exome sequencing, APC, MUTYH, WTX

## Abstract

Familial adenomatous polyposis (FAP) and MUTYH‐associated polyposis (MAP) are inherited disorders associated with multiple colorectal adenomas that lead to a very high risk of colorectal cancer. The somatic mutations that drive adenoma development in these conditions have not been investigated comprehensively. In this study we performed analysis of paired colorectal adenoma and normal tissue DNA from individuals with FAP or MAP, sequencing 14 adenoma whole exomes (eight MAP, six FAP), 55 adenoma targeted exomes (33 MAP, 22 FAP) and germline DNA from each patient, and a further 63 adenomas by capillary sequencing (41 FAP, 22 MAP). With these data we examined the profile of mutated genes, the mutational signatures and the somatic mutation rates, observing significant diversity in the constellations of mutated driver genes in different adenomas, and loss‐of‐function mutations in WTX (9%; p < 9.99e‐06), a gene implicated in regulation of the WNT pathway and p53 acetylation. These data extend our understanding of the early events in colorectal tumourigenesis in the polyposis syndromes. © 2015 The Authors. *The Journal of Pathology* published by John Wiley & Sons Ltd on behalf of Pathological Society of Great Britain and Ireland.

## Introduction

Over the last three decades there has been a dramatic improvement in our understanding of the genetic basis of germline susceptibility to colorectal cancer (CRC) 1. This began with the identification of the adenomatous polyposis coli gene (*APC*) 2, in which germline mutations cause familial adenomatous polyposis (FAP), followed by the discovery of other genes such as *MSH2* in Lynch syndrome 3 and mutY homologue (*MUTYH* ) in *MUTYH*‐associated polyposis (MAP) 4 (also called MYH‐associated polyposis). Germline variants in the genes *LKB1*, *SMAD4*, *GREM1*, *PTEN*, *BMPR1A* and *AXIN2* have also been implicated in predisposition to colorectal cancer and highlight a role for many pathways in tumourigenesis of the colon 5. More recently, germline variants in the exonuclease domains of the polymerase‐ϵ catalytic subunit gene (*POLE*) and in the DNA polymerase delta catalytic subunit (*POLD1*) gene have been linked to colorectal adenoma and carcinoma development 6. Variants in *POLE* and *POLD1* dramatically increase the somatic mutation rate, resulting in C:G → T:A somatic base changes 7. While the majority of colorectal cancers are sporadic, variants in the above‐mentioned genes, and in several other high‐penetrance susceptibility genes including *MLH1*, *MSH6* and *PMS2*, collectively account for around 5% of all cases 5. While colorectal cancer is a common endpoint of germline variants in these genes, they initiate tumourigenesis in different ways, meaning that the landscape of somatic mutations, the genes that are mutated and the paths to malignancy, are likely to differ. As a corollary, the multiplicity of tumours in patients with germline mutations in these genes differ and clinical outcomes and responses to therapy can vary, suggesting a complex interplay between the germline genetics of each patient and the somatic landscape of adenomas and tumours that develop within the bowel 8. Despite major initiatives to analyse sporadic colorectal cancers 9, little is known about the somatic landscape of tumours from patients with hereditary forms of the disease. Here we set out to profile somatic mutations in pre‐malignant adenomas in two hereditary colon cancer syndromes, FAP and MAP.

In FAP, adenomas may develop following somatic inactivation of the wild‐type allele of *APC*, an event that is thought to be among the earliest somatic changes occurring during tumourigenesis in these patients 10. APC normally binds to GSK3β as part of a complex called the ‘destruction complex’ which regulates β‐catenin stability, and hence the output of the WNT pathway 11. Loss or attenuation of the activities of the destruction complex results in elevated levels of β‐catenin and of downstream effectors such as CCND1, AXIN2 and BIRC5 12. These proteins participate in the cell cycle, growth and regulation of cell death, respectively. The location of the germline mutation in *APC* in a FAP patient and the mode by which the wild‐type allele of the gene is inactivated during adenomagenesis influence the degree to which the WNT pathway is activated 13. The level of WNT pathway activation influences the multiplicity of intestinal polyposis and the growth of the adenomas that form, and is described by the ‘just‐right hypothesis’, which suggests that adenomas aim to have sufficient WNT activation to drive cell growth without tipping cells into apoptosis or evoking cell death 14. Fine‐tuning of the WNT pathway is thus central to colorectal tumourigenesis 15. MutY homologue (*MUTYH*) is a DNA glycosylase that removes adenines mis‐incorporated opposite 8‐oxo‐7,8‐dihydro‐2′‐deoxyguanosine (8‐oxodG) 16. Patients with MAP carry bi‐allelic loss‐of‐function mutations in the *MUTYH* gene, which in targeted sequencing studies of adenomas was found to manifest as an increase in somatic G:C → T:A mutations at the *APC* locus 17. Loss of *MUTYH* by itself is not oncogenic, akin to some other colorectal cancer syndromes, such as Lynch syndrome. With the exception of the aforementioned mutations in *APC*, transversions in *RAS* resulting in the generation of a G12C amino acid change are the only other established somatic events in *MUTYH*‐driven tumourigenesis. The landscape of somatic changes, the rate of somatic mutation and the genes that are mutated in this disease are unknown.

Several studies have used next‐generation sequencing of human colorectal cancers to survey their somatic mutational landscape. The *Cancer Genome Atlas* (TCGA) characterized the genomes of 276 sporadic colorectal cancers, focusing almost exclusively on invasive cancers and metastatic tumours 9. Other studies have analysed the exomes of microsatellite‐instable (MSI) primary cancers 18, while Nikolaev *et al* 19 performed a detailed and comprehensive analysis of 24 sporadic adenomas, revealing a signature of deamination (C → T at CpG sites), suggesting a role for replication stress in mutational acquisition. Here we focus on the early evolution of adenomas from MAP and FAP patients and investigate the somatic mutation rate and the pattern of mutation. We also identify mutated driver genes, including truncating mutations in *WTX* (also known as *FAM123B* and *AMER1*).

## Materials and methods

### Tumour collection

Ethical approval and written informed consent from each participant was obtained under UK NHS Research Ethics Committee approvals 02/09/22 and 12/WA/0079. Adenomas were harvested at colectomy or polypectomy from the colorectum of patients with confirmed germline mutations in *APC* or *MUTYH*. Larger adenomas were halved longitudinally, with one part being snap‐frozen in liquid nitrogen and the other formalin‐fixed for histopathology. Smaller lesions were snap‐frozen and histopathology performed using a small sample cut from the frozen material. A description of the germline mutations carried by all patients and a summary of their clinical histories is available in Table S1 (see supplementary material). Three sets of adenomas were analysed, using either whole‐exome sequencing, targeted‐exome sequencing or capillary sequencing of *WTX*, as described below. Histopathological analysis of adenomas was performed by a clinical gastrointestinal histopathologist (GTW). A summary of the pathology reports is provided in Table S2 (see supplementary material). DNA was extracted using the Qiagen DNeasy Kit. Lesions selected for analysis were of similar (sub‐cm) size, with most showing low‐grade dysplasia.

### Whole‐exome and targeted‐exome sequencing

**Figure 1:**
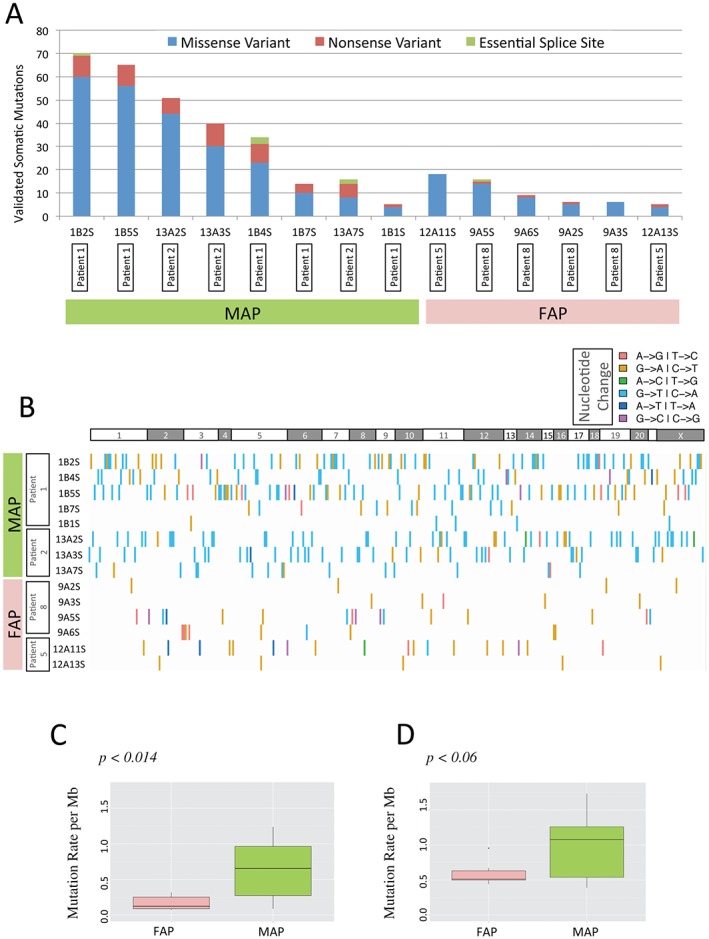
Somatic mutation calls from adenomas from patients with MAP or FAP. (A) Validated protein‐changing somatic mutation calls from MAP and FAP adenomas: missense, nonsense and splice site variants are shown. (B) Profile of the validated somatic calls for the variants shown in (A): the type of nucleotide change is indicated by the colour of each bar for each patient; adenoma IDs for each patient are shown on the y axis (for clinical details, see supplementary material, Tables S1, S2); horizontal white/grey bar denotes chromosomes (no mutations were found on chromosome 21). (C) Box plots showing, per megabase (Mb), median and 25th and 75th percentiles of validated somatic variant calls calculated from the data shown in (A). (D) Box plots showing, per megabase (Mb), median and 25th and 75th percentiles of somatic variant calls made using the Cake pipeline 21; p values represent the results of Student's two‐tailed t‐test. The variant calls used in Figure 1 are provided in Table S3.

Whole‐exome sequencing was performed using the Agilent whole‐exome capture kit (SureSelectXT Human All Exon 50 Mb), as described previously 20. Captured material was indexed and sequenced on the Illumina platform at the Wellcome Trust Sanger Institute. Targeted capture sequencing was performed using baits designed against the genes *APC*, *WTX/FAM123B*, *ATRNL1*, *BCL9L*, *BRCA1*, *BRCA2*, *CXCR5*, *DMD*, *FBXW7*, *GPR112*, *HUWE1*, *KMT2C/MLL3*, *NF1*, *PTEN*, *SLFN5*, *SMAD4*, *SORCS1*, *TP53*, *UBR2* and *ZNF37A*, selected for sequencing on the basis of being recurrently mutated, or truncated, in the unfiltered whole‐exome sequencing data; these genes are also enriched for non‐silent mutations in the genome sequencing of sporadic CRC 9. A breakdown of the sequencing metrics for each sample is provided in Figure S1 (see supplementary material). We collected and whole‐exome sequenced 14 adenomas (eight MAP and six FAP) from two MAP patients and two FAP patients, and corresponding germline control DNA for each patient (Figure 1; see also supplementary material, Figure S1, Table S2). In a similar way, 22 FAP and 33 MAP adenomas and corresponding blood leukocyte DNA from three FAP and four MAP patients were sequenced using the targeted bait set (see supplementary material, Table S2). DNA from all of the adenomas and corresponding leukocyte controls was available for follow‐up genotyping/validation. Seven cases that were whole‐exome sequenced were also targeted‐exome sequenced (four FAP and three MAP) to compare these platforms (see supplementary material, Table S2). Table 1 provides a summary of the sequenced samples.

**Table 1:** Samples used as part of this study

Patient No.	Cohort	Number of adenomas		
		Whole‐exome sequencing (WES)	Targeted‐gene sequencing (TGS)	Capillary sequencing
1	MAP	5	4	4
2	MAP	3	5	
3	MAP		3	
4	MAP		21	14
5	FAP	2		5
6	FAP		10	
7	FAP		11	
8	FAP	4	1	
9	FAP			5
10	MAP			4
11	FAP			2
12	FAP			5
13	FAP			17
14	FAP			7
Total		14	55	63

Seven samples were sequenced by both whole‐exome and targeted‐exome sequencing but are not shown here; details are available in Table S2 (see supplementary material).

### Somatic single nucleotide variant calling

DNA sequence data from paired adenoma/normal constitutional DNA samples were presented to the Cake pipeline, which uses the somatic variant callers Bambino, CaVEMan, SAMtools mpileup and VarScan2 21. As described previously 21, we used a somatic caller merging approach to identify somatic variants, selecting only those detected using three or more of these algorithms for further analysis. We have previously shown that this approach increases the sensitivity and specificity of variant detection 21. These calls were further filtered using modules such as the single nucleotide polymorphisms (SNPs) filter, excluding 1000 Genomes Project phase 1 variants with minor allele frequencies greater than 0.01 (> 0.01), and by standard variant filtering.

### Variant validation by Sequenom

We attempted to validate all non‐silent somatic variant calls from both the targeted‐ and whole‐exome sequencing experiments using the Sequenom platform. Both normal tissue and adenoma DNA samples were analysed as described previously 22.

### Capillary sequencing of WTX and KRAS

*WTX* was one of several genes found to carry truncating mutations and was capillary sequenced in a larger panel of adenomas (41 FAP and 22 MAP; see supplementary material, Table S2) to extend the data collected from the whole‐exome and targeted‐exome sequencing experiments. In brief, primers were designed against each exon of the gene, amplicons were bidirectionally sequenced and variants called using Mutation Surveyor Software, followed by manual inspection. *KRAS* sequencing was performed as described by Jones *et al* 23.

### Mutation signature analysis

We interpreted the mutational catalogue of MAP and FAP using validated variants called from the whole‐exome sequence data, and also the raw variant calls made by the Cake pipeline. For this task we used EMu, a probabilistic algorithm that infers the number of mutational processes operative and their individual signatures 24. Mutations were mapped to the 96 possible trinucleotide combinations, taking into account the possibility for each mutation to occur in the context of each trinucleotide type within the human genome. As the model underlying EMu assumes that the input samples are independent, we further collapsed the mutation data by patient and performed a patient‐centric signature analysis.

### Statistical analysis of WTX mutations

To determine whether *WTX* was significantly enriched for nonsense mutations, we used Monte Carlo simulations. 100 000 iterations were generated, where six nucleotide changes were randomly introduced into the *WTX* sequence (six being the number of changes found in *WTX* in the targeted sequencing experiment; five nonsense and one synonymous), using the underlying base change probability from TCGA data across all tumour types. We then computed all possible outcomes for these mutations from each iteration and compared these frequencies to the frequency of truncating mutations found in the targeted‐exome analysis.

## Results

### Calling and validation of somatic variants

We attempted to validate all non‐silent positions at which a candidate somatic variant call had been made from the whole (573) or targeted (45) exome data using the Sequenom platform. To do this, we genotyped DNA from each adenoma and a matched normal tissue or leukocyte control DNA sample. We successfully designed assays against 434/573 positions from the whole‐exome sequencing experiment and 42/45 positions from the targeted‐exome sequencing experiment. The overall validation rate for the 434 calls from the whole‐exome sequencing of the MAP/FAP polyps was 80.87% (351 successfully genotyped somatic SNVs). The validation rate for the targeted‐exome experiment was 95.23% (40/42) (see supplementary material, Tables S3, S4).

### The frequency and distribution of somatic mutations in FAP and MAP adenoma exomes

Figure 1A shows the breakdown of Sequenom‐validated protein‐changing or disruptive somatic variants by adenoma and disease and their mutational class; missense, nonsense or essential splice site. Figure 1B shows the breakdown of validated non‐silent somatic variants by mutational class, disease, tumour and patient. All variant calls, including synonymous variants, are shown in Figure S2A, and their mutational profile is shown in Figure S2B (see supplementary material); variant validation metrics are provided in Figure S3. Analysis of the somatic mutational landscape of protein‐changing variants in this way revealed a mean somatic mutational burden of 0.65 mutations/Mb in MAP adenomas compared to 0.16/Mb in FAP adenomas (*p <* 0.014) (Figure 1C). When using the raw output of the Cake pipeline, there were 0.98 and 0.59 variants/Mb for MAP and FAP, respectively (*p <* 0.06) (Figure 1D). These findings suggest an increased mutational burden resulting from loss of *MUTYH* activity in the range of 1.5–4.0‐fold, comparable to findings reported in a recent study of lymphoblastoid cell lines established from MAP patients 25. Importantly, we observed a significant increase (*p <* 0.012; Student's two‐tailed *t‐*test) in the proportion of truncating mutations found in adenomas from MAP patients compared to those from FAP patients (Figure 1A). This observation may reflect the different profiles of chromosomal imbalances in FAP and MAP adenomas 26, such that tumour suppressor genes undergo allelic loss in FAP rather than being disrupted by point mutations.

### The mutational signatures of FAP and MAP

**Figure 2:**
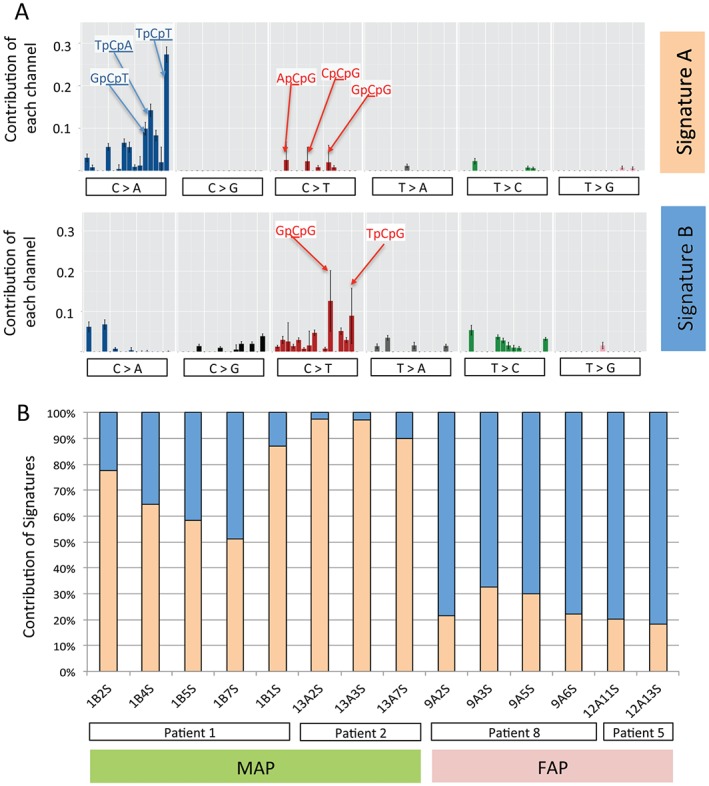
Mutational signatures in MAP and FAP. (A) The mutation spectra across 96 mutational channels (each representing a trinucleotide context, as described previously 37). (B) Mutational signature activity plot, indicating the proportion of somatic mutations found in adenomas from MAP and FAP patients that can be attributed to either signature A or signature B; the validated positions in Table S3 (see supplementary material) were used for this analysis

We determined the pattern and distribution of somatic nucleotide changes found in the eight MAP and six FAP adenomas that were whole‐exome sequenced. Using both validated variant calls (Figure 1) and the output of the Cake pipeline (see supplementary material, Figure S2, Table S3), we used EMu software to discern mutational signatures as a way of identifying mutational processes that may be operative. This analysis revealed strong statistical support [Δ Bayesian information criterion (Δ‐BIC) score >171] for two distinct mutational processes, signatures A and B, when all 573 variant positions were used (see supplementary material, Figure S4). Analysis of validated calls assuming that two signatures are present led to similar results (Figure 2A). Both signatures include C → T mutations at XpCpG sites, compatible with spontaneous deamination, but signature A also included C → A mutations, especially at TpCpX sites. The latter may result from sequence motifs that have enhanced mutability. We also used EMu to estimate the mutational composition of the 14 individual adenomas with respect to the two mutational processes (Figure 2B). Signature A, which is composed primarily of C:G → A:T transversions that are typically associated with the failure to remove misincorporated adenines opposite 8‐oxo‐7,8‐dihydro‐2′‐deoxyguanosine (8‐oxodG), was the dominant signature in MAP adenomas (Figure 2). In contrast the dominant mutational signature in FAP adenomas was signature B (Figure 2B).

### Driver mutations in MAP and FAP adenomas

**Figure 3:**
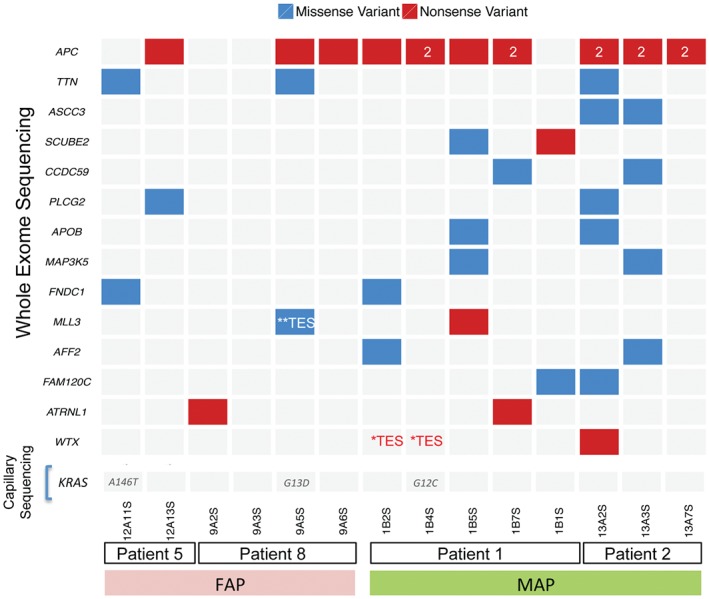
Candidate driver genes in adenomas from MAP and FAP patients. The most frequently mutated genes in MAP and FAP adenomas are shown: red boxes, nonsense mutations; blue boxes, missense mutations; a number in a variant box indicates where multiple mutations of the same class are found. KRAS mutational status, determined by capillary sequencing, is also shown; some of the adenomas were also sequenced by targeted‐exome sequencing (TES); ^**^ TES, missense mutations; ^*^ TES, nonsense mutations (see supplementary material, Table S2). All positions were validated by Sequenom genotyping of tumour and control DNA; grey indicates no mutation found

**Figure 4:**
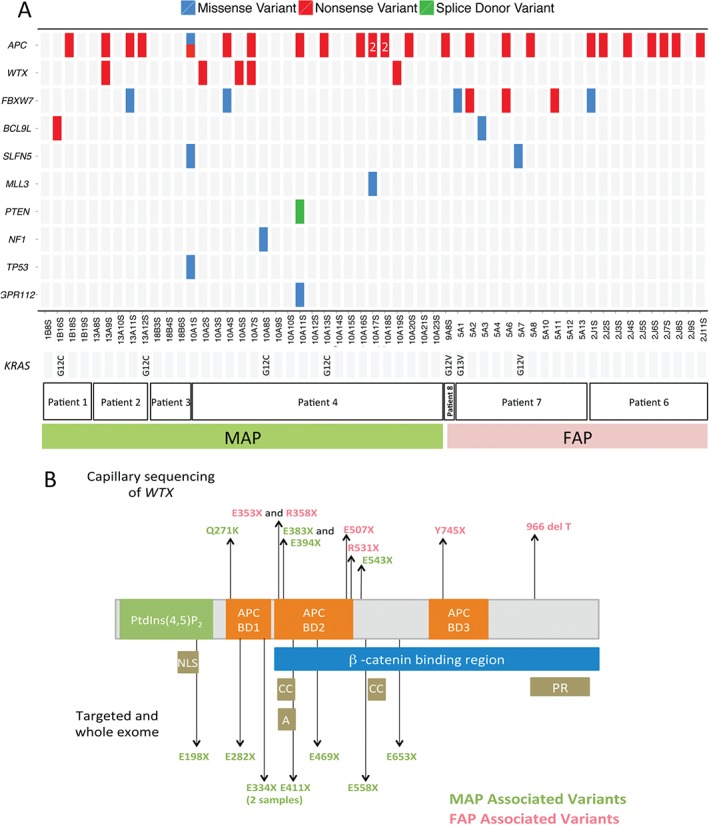
WTX is a driver gene in adenomas in MAP and FAP. (A) The validated somatic mutations identified by targeted resequencing of 33 MAP and 22 FAP adenomas are shown (see supplementary material, Table S4): red, nonsense somatic mutations; blue, missense variants; green, essential splice site variants; KRAS mutations, which were assessed by capillary sequencing, are also shown; grey indicates no mutation found. (B) Somatic variants identified in WTX by whole‐ and targeted‐exome sequencing (bottom) and capillary sequencing (top): the protein domains and positions are derived from Ensembl (ENST00000330258); NLS, nuclear localization signal; AA, acidic region; CC, coiled‐coil domain; PR, proline‐rich region; BD, binding domain

We used the 351 validated somatic protein changing variants from exome sequencing (see supplementary material, Table S3) to ask which genes are likely to be driver genes in colorectal adenoma formation. Of the six FAP adenomas, three had somatic nonsense variants in *APC*, all falling into the β‐catenin binding domains of APC and distal to the germline *APC* mutation found in these patients (Figure 3). Of the eight MAP adenomas, five had bi‐allelic nonsense *APC* mutations. Truncating mutations were also validated in *SCUBE2*, *RELN*, *FBXW7*, *MLL3*, *WTX/FAM123B*, *OTUD7B* and *KPRP* across the MAP and FAP adenomas (see supplementary material, Table S3). Two adenomas (Figure 3) were found to carry truncating mutations in the attractin‐like 1 (*ATRNL1*) gene. Known driver genes from the cancer gene census in which we identified missense mutations included *MAP3K5* and *NRAS* (p.Q61K; Figure 3; see also supplementary material, Table S3). Two adenomas carried missense mutations in the phospholipase C, γ2 gene (*PLCG2*), which is related to *PLCG1* recently described as a driver gene in angiosarcoma 27. We also found three adenomas carrying protein‐changing *KRAS* mutations; MAP polyp 1B4S carried a p.G12C, while FAP polyps 12A11S and 9A5S carried p.A146T and p.G13D changes, respectively. All of these variants were validated by capillary sequencing. We next designed a custom capture bait set against genes identified as carrying truncating mutations, or as being recurrently mutated in the unfiltered whole‐exome sequencing data (see Materials and methods). Analysis of 55 adenomas (33 MAP and 22 FAP) and corresponding control DNA using this bait set yielded the Sequenom‐validated somatic mutations shown in Figure 4A. Overviews of the somatic mutations called by the Cake pipeline, the results of their validation by Sequenom genotyping and their mutational profiles are shown in the supplementary material (see supplementary material, Figure S5, Table S4). Of particular note was the identification of truncating mutations in *WTX* and mutations in genes such as *TP53*, *FBXW7* and *PTEN*. Four MAP polyps were found to carry canonical *KRAS* p.G12C mutations resulting from a somatic G:C → T:A change; GGT → TGT, a figure in accord with a previous report 23. Several adenomas that were whole‐exome sequenced were also targeted‐exome sequenced (Table 1; see also supplementary material, Tables S2, S4). Owing to the extremely high depth of sequence coverage obtained in the targeted‐exome studies, we identified and validated additional driver mutations not seen by whole‐exome sequencing (Figure 2; see also supplementary material, Table S4).

### WTX mutations in FAP and MAP

During the targeted sequencing of 33 MAP and 22 FAP adenomas, we identified and validated five truncating *WTX* mutations, all in MAP lesions, representing a statistically significant enrichment of truncating mutations in this gene (*p <* 9.99e‐06). *WTX* mutations have been reported in advanced colorectal cancer, but their role in early stages of colorectal tumourigenesis is unknown. In order to determine whether there were differences in the frequency or profile of *WTX* mutations between early MAP‐ and FAP‐associated adenomas, we employed capillary sequencing to screen the exons and exon–intron boundaries of *WTX* in a further 22 MAP and 41 FAP adenomas. We identified nine further truncating mutations (including one frameshift mutation), six in FAP adenomas and three in MAP adenomas, and one missense mutation in a MAP adenoma (Figure 4B; see also supplementary material, Table S5).

**Figure 5:**
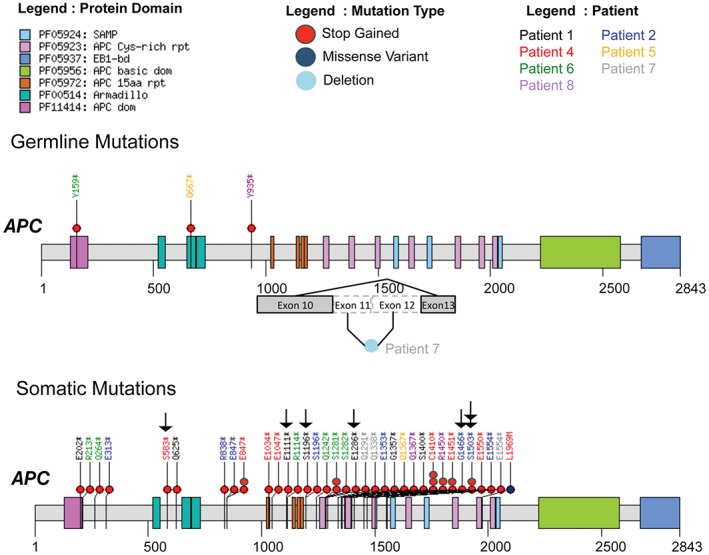
Germline and somatic APC mutations and somatic WTX mutational status. Predicted consequences of germline and somatic APC mutations found in adenomas from FAP and MAP patients are shown. Colour‐coded circles indicate mutation type. Ensembl APC protein isoform ENST00000257430 and colour coded protein domains are shown: FAP, patients 5–8; MAP, patients 1, 2 and 4; mutations are colour‐coded by patient; arrows, lesions also carrying truncating WTX mutations identified by next‐generation sequencing (see Figures 2, 4). Where an adenoma was found to carry bi‐allelic APC mutations, we indicated the presence of a truncating WTX mutation against both mutations. We sequenced and validated somatic APC mutations in two adenomas from patient 2 that resulted in an amino acid change, p.S1503 ^*^. Both of these adenomas also carried truncating somatic WTX mutations (at positions p.E411X and p.E558X)

The 17 truncating mutations of *WTX* identified in the different phases of our study are all likely to impact the function of its β‐catenin binding region (Figure 4B). Although *WTX* is on the X chromosome, we identified mutations in adenomas from both male and female patients. We did not observe somatic bi‐allelic mutations in adenomas from females, suggesting that Lyonization may be responsible for loss of *WTX* function. *WTX* was originally identified as a gene involved in the development of Wilms' tumour of the kidney 28 and has reported roles in the regulation of the WNT pathway, TP53 and cell fate, and in the localization of the tumour suppressor protein WT1 29, 30, 31. Germline truncating mutations in *WTX* have also been linked to a sclerosing skeletal dysplasia (OSCS; MIM300373) and are not considered to be associated with an increased risk of tumours, although relatively early‐onset colorectal cancer occurred in one of 25 adult patients in the original report 32. Mass‐spectrometry studies have revealed that WTX forms a complex with β‐catenin, AXIN1, β‐transducin repeat‐containing protein 2 (β‐TrCP2) and adenomatous polyposis coli (APC) to promote the ubiquitination and degradation of β‐catenin 33. Knockdown experiments have shown WTX to be a negative regulator of the WNT pathway 33; thus, mutation of *WTX* may result in activation of this pathway and the promotion of tumourigenesis. Figure 5 shows the location of the somatic *APC* mutations identified in each adenoma and their *WTX* mutational status. The majority of the *APC* mutations found in adenomas with *WTX* mutations fell into the β‐catenin or mutator cluster region (MCR), potentially resulting in impaired formation of the destruction complex, rather than its complete loss 34, a scenario that may allow further modulation of β‐catenin signalling via changes in WTX expression.

## Discussion

In this study we explored the somatic mutational landscape of early‐stage premalignant adenomas from patients with germline mutations in *APC* and *MUTYH* as a first step towards defining the catalogue of mutated genes. This task is essential for identifying which sets of mutations are most likely to lead adenomas to progress to colorectal cancer. We revealed that MAP adenomas have approximately two to four times the number of coding region somatic mutations when compared to FAP adenomas, and that these mutations are overwhelmingly G:C → T:A mutations, in keeping with the expected signature associated with *MUTYH* loss. This observation confirms, for the first time, the expectation that deficiency of *MUTYH* leads to a mutator phenotype in colorectal tumours, and is consistent with the observation of substantial colorectal cancer risk in MAP, even in the absence of dense polyposis 35. We find significant complexity in the patterns of mutated genes, such that, with the exception of *APC*, *KRAS* and *WTX* mutations, few adenomas have the same set of mutated driver genes, a novel observation that may have implications for the definition of high‐risk adenomas in the era of molecular pathology.

In FAP and MAP we found that 50% of tumours carried somatic *APC* mutations (in the whole‐ and targeted‐exome experiments combined). Notably, the frequency of bi‐allelic mutations was found to differ between our whole‐exome and targeted‐exome studies, despite an identical ascertainment of samples. In those lesions in which we did not find loss‐of‐function mutations in *APC* it is possible that the gene is disrupted by an imbalance of chromosome 5 26 or by copy number neutral changes at the *APC* locus 36. The number of *APC* mutations we observed here was slightly lower than estimates from the TCGA, who report frequencies of 60–80% for hypermutated and non‐hypermutated cancers, respectively, and probably reflects the difficulties of identifying somatic mutations in lesions with low tumour cellularity. Intriguingly, across all protein‐coding genes we observed a significant enrichment for truncating mutations in MAP adenomas compared to FAP (*p <* 0.012). We also observed a significant (*p <* 9.99e‐06) incidence of truncating *WTX* mutations, with only *APC* being more frequently mutated. Our study demonstrated that in patients with MAP or FAP diverse molecular mechanisms are operational, even at early stages of colorectal tumourigenesis. This suggests that medical therapies for these disorders may be most effective if they target the initiating events of tumourigenesis prior to the development of mutationally diverse adenomas.

## Author contributions

MR, AF, AGR and VM performed computational analysis; CHW, JT, PS, SI and JM performed laboratory experiments and contributed to the analysis; GTW performed histopathological analysis; and MR, CHW, JRS and DJA designed the experiments and wrote the paper.

**Supplementary material on the internet**

The following supplementary material may be found in the online version of this article:

**Figure S1.** Sequencing metrics for the sequencing of adenomas and matched normal tissue samples from patients with MAP or FAP

**Figure S2.** Somatic variant calls made by the Cake pipeline from MAP and FAP cases

**Figure S3.** Results of Sequenom validation experiments of somatic variant calls made using Cake against adenoma/matched normal tissue pairs from MAP and FAP cases

**Figure S4.** Mutational signatures in MAP and FAP using all 573 calls made by the Cake pipeline

**Figure S5.** Results of targeted sequencing of adenoma/matched normal tissue pairs from MAP and FAP patients

**Table S1.** A summary of the clinical details of each patient

**Table S2.** A summary of the samples sequenced as part of this study

**Table S3.** Whole‐exome variant calls

**Table S4.** Targeted‐exome variant calls

**Table S5.** Capillary sequencing of *WTX* and variant calls

## Supporting information

Sequencing metrics for the sequencing of adenomas and matched normal tissue samples from patients with MAP or FAP. (A) Net mapped bases for all samples sequenced as part of this study: orange bars denote normal/germline control data; blue bars denote adenoma data; y axis shows the net mapped bases in gigabases (Gb) for each sample. (B) Net mapped bases for targeted‐exome sequencing of MAP and FAP adenomas and matched normal tissue DNA; y axis shows the net mapped bases (Gb) for each sample. (C, D) The comparative read depth at positions that were successfully validated from the whole‐exome or targeted‐exome sequencing data shown in (A) or (B), respectively; in (C, D) the median and 25th and 75th percentiles are shown

Somatic variant calls made by the Cake pipeline from MAP and FAP cases. (A) The number of somatic variant calls made per adenoma/matched normal tissue pair: variants are broken down by class – missense, nonsense, essential splice site, miRNA variant and synonymous variant. (B) Landscape of somatic changes called by the Cake pipeline, using the three of four caller approaches outlined in Materials and methods [21]

Results of Sequenom validation experiments of somatic variant calls made using Cake against adenoma/matched normal tissue pairs from MAP and FAP cases. (A) The overall validation success rate, broken down by the reason why a variant failed to validate. (B) A stepwise view of the validation success rate and reasons why a variant failed to validate

Mutational signatures in MAP and FAP using all 573 calls made by the Cake pipeline. (A) The mutation spectra across 96 mutational channels (each channel represents a trinucleotide context, as described previously) [37]. (B) Mutational signature activity plot, indicating the proportion of somatic mutations found in adenomas from MAP and FAP patients that can be attributed to either signature A or signature B

Results of targeted sequencing of adenoma/matched normal tissue pairs from MAP and FAP patients. (A) The successfully validated variants called by Cake and validated by Sequenom genotyping are shown: sample IDs are shown on the y axis, chromosome positions on the x axis; colours within the plot denote base changes. (B) Validated somatic mutations displayed in (A) by adenoma/matched normal tissue pairs: blue, missense; red, nonsense; green, essential splice site variants. (C) Comparative read depths of successfully genotyped variants from the targeted sequencing of FAP and MAP adenomas; median and 25th and 75th percentiles are shown

A summary of the clinical details of each patient

A summary of the samples sequenced as part of this study

Whole‐exome variant calls

Targeted‐exome variant calls

Capillary sequencing of WTX and variant calls
