# Supplementary figures and images for: Adenoma development in familial adenomatous polyposis and MUTYH‐associated polyposis: somatic landscape and driver genes
Source: J Pathol. 2015 Nov 2;238(1):98–108. doi: 10.1002/path.4643 (PMC4832337; doi:10.1002/path.4643)

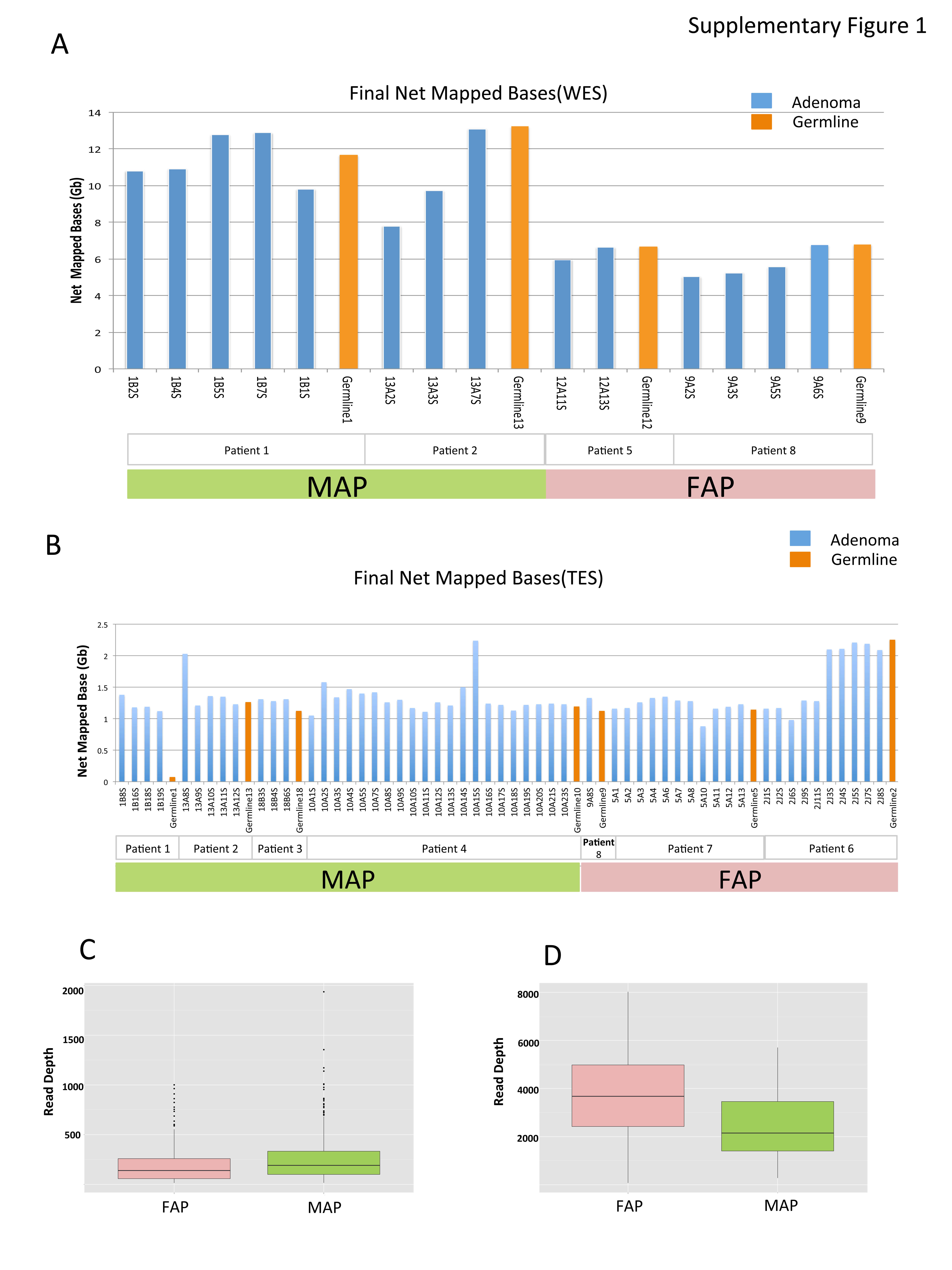

Supplement: Supplementary file 1 — Sequencing metrics for the sequencing of adenomas and matched normal tissue samples from patients with MAP or FAP. (A) Net mapped bases for all samples sequenced as part of this study: orange bars denote normal/germline control data; blue bars denote adenoma data; y axis shows the net mapped bases in gigabases (Gb) for each sample. (B) Net mapped bases for targeted‐exome sequencing of MAP and FAP adenomas and matched normal tissue DNA; y axis shows the net mapped bases (Gb) for each sample. (C, D) The comparative read depth at positions that were successfully validated from the whole‐exome or targeted‐exome sequencing data shown in (A) or (B), respectively; in (C, D) the median and 25th and 75th percentiles are shown [file PATH-238-98-s001.tiff]

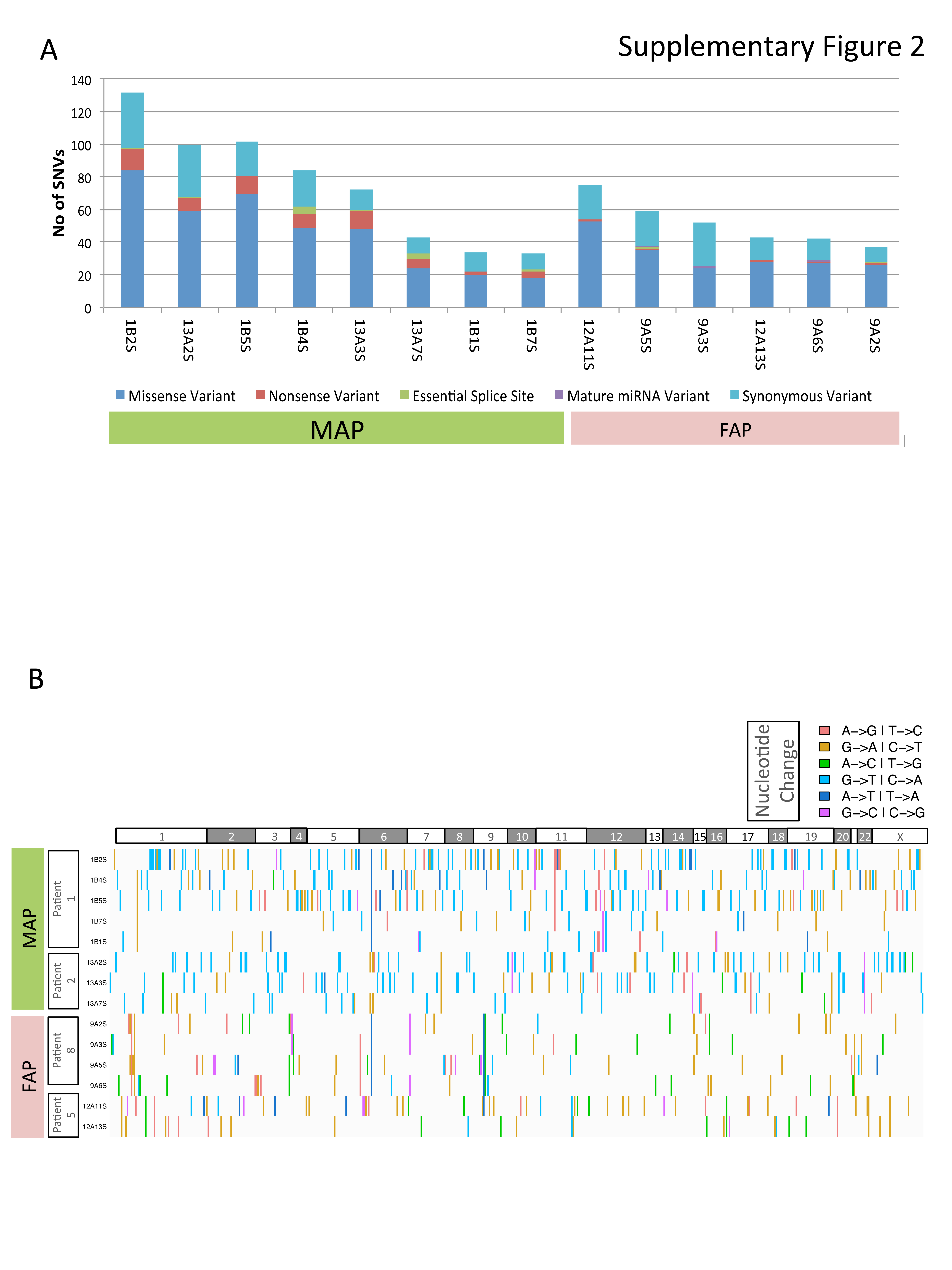

Supplement: Supplementary file 2 — Somatic variant calls made by the Cake pipeline from MAP and FAP cases. (A) The number of somatic variant calls made per adenoma/matched normal tissue pair: variants are broken down by class – missense, nonsense, essential splice site, miRNA variant and synonymous variant. (B) Landscape of somatic changes called by the Cake pipeline, using the three of four caller approaches outlined in Materials and methods [21] [file PATH-238-98-s002.tiff]

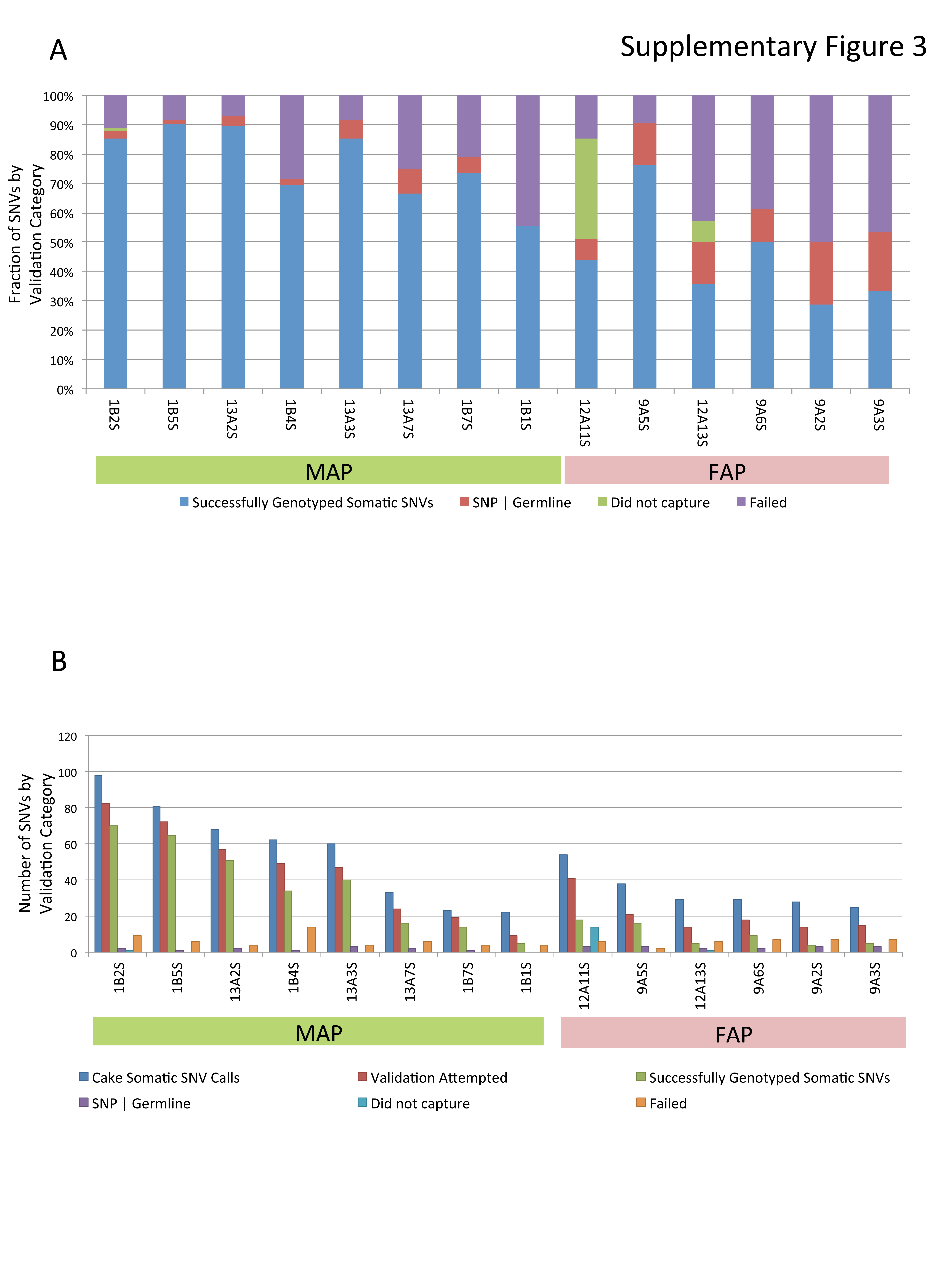

Supplement: Supplementary file 3 — Results of Sequenom validation experiments of somatic variant calls made using Cake against adenoma/matched normal tissue pairs from MAP and FAP cases. (A) The overall validation success rate, broken down by the reason why a variant failed to validate. (B) A stepwise view of the validation success rate and reasons why a variant failed to validate [file PATH-238-98-s003.tiff]

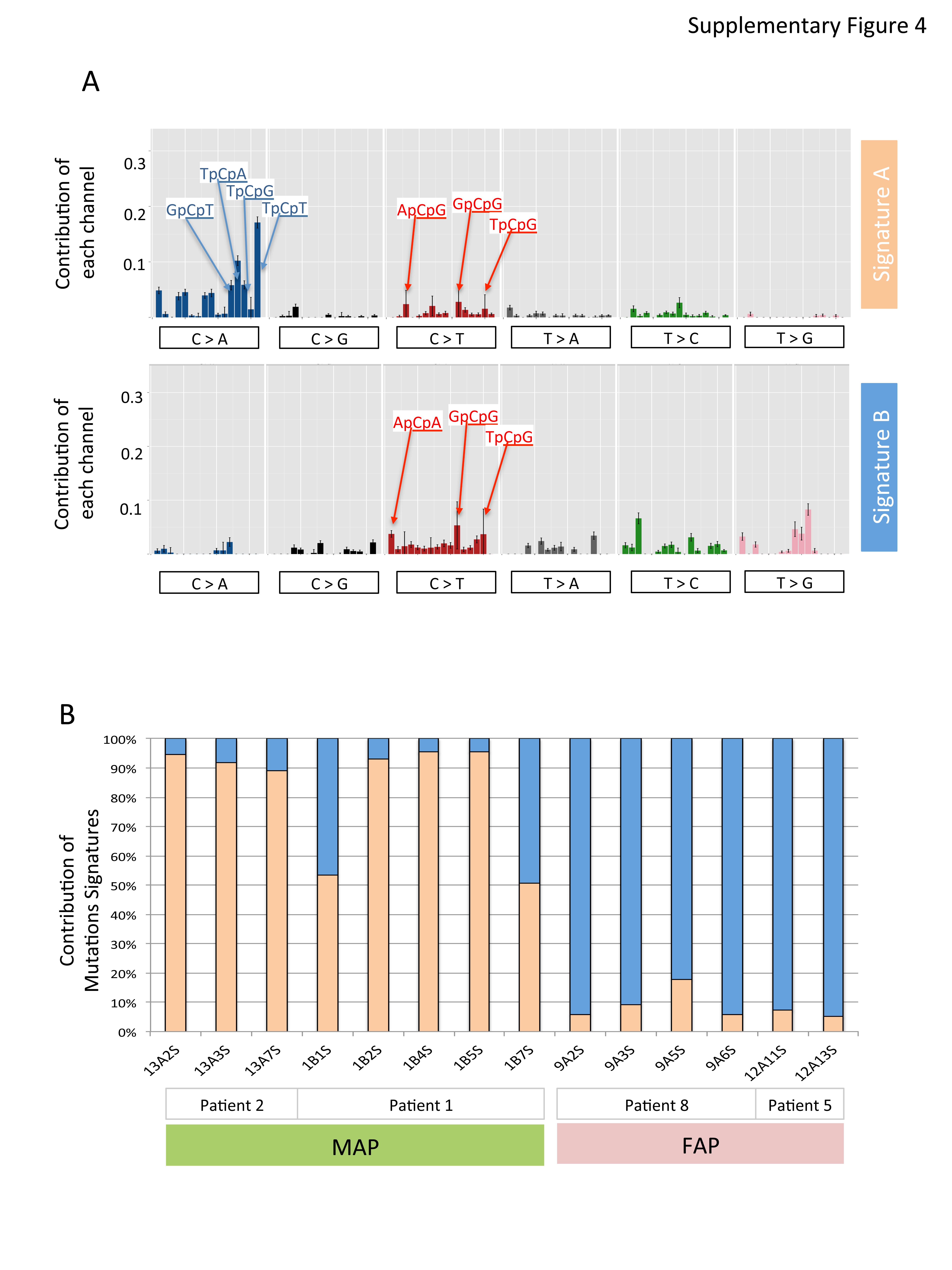

Supplement: Supplementary file 4 — Mutational signatures in MAP and FAP using all 573 calls made by the Cake pipeline. (A) The mutation spectra across 96 mutational channels (each channel represents a trinucleotide context, as described previously) [37]. (B) Mutational signature activity plot, indicating the proportion of somatic mutations found in adenomas from MAP and FAP patients that can be attributed to either signature A or signature B [file PATH-238-98-s004.tiff]

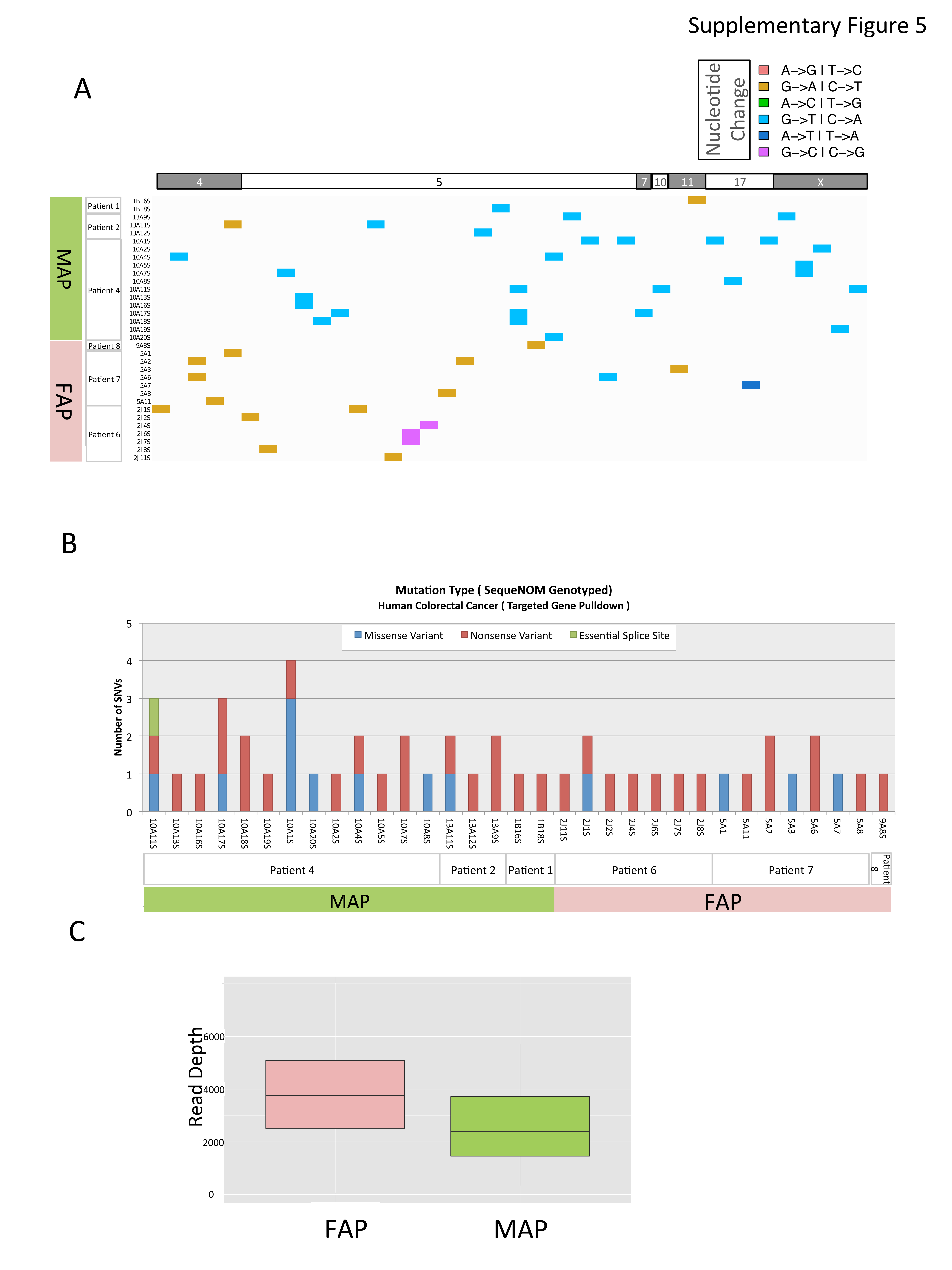

Supplement: Supplementary file 5 — Results of targeted sequencing of adenoma/matched normal tissue pairs from MAP and FAP patients. (A) The successfully validated variants called by Cake and validated by Sequenom genotyping are shown: sample IDs are shown on the y axis, chromosome positions on the x axis; colours within the plot denote base changes. (B) Validated somatic mutations displayed in (A) by adenoma/matched normal tissue pairs: blue, missense; red, nonsense; green, essential splice site variants. (C) Comparative read depths of successfully genotyped variants from the targeted sequencing of FAP and MAP adenomas; median and 25th and 75th percentiles are shown [file PATH-238-98-s005.tiff]
