# Supplementary material for: Adenoma development in familial adenomatous polyposis and MUTYH‐associated polyposis: somatic landscape and driver genes
Source: J Pathol. 2015 Nov 2;238(1):98–108. doi: 10.1002/path.4643 (PMC4832337; doi:10.1002/path.4643)
Supplement: Supplementary file 6 — A summary of the clinical details of each patient [file PATH-238-98-s006.docx]

**Table S1**

**Patient and Sample Details**

1. **MAP Patients**

**[Patient 1]** 1B* polyps *MUTYH* c.536A>G (p.Tyr179Cys), c.1187G>A (p.Gly396Asp)

Female. Identified with approx. 10 polyps that were removed at 51 years of age at colonoscopy undertaken because of family history of CRC. Further colonoscopy at 55 years showed at least 20 polyps. Colectomy at 56 years confirmed approx. 50 adenomas and 3 hyperplastic polyps distributed throughout colorectum. Polyps 1B1-1B7 taken at 55 years. Polyps 1B1S to 1B23S taken at 56 years of age. Otherwise well.

**[Patient 2]** 13A* polyps *MUTYH* c.303C>G (p.Tyr101X) homozygous

Female. Identified with >50 polyps of > 5mm and innumerable smaller polyps throughout colon at colonoscopy at 38 years of age, following genetic diagnosis of MAP. Rectum showed relative sparing. MAP genetic testing was undertaken because of family history of polyposis. All polyps for this study taken at colectomy at 39 years of age. Otherwise well.

**[Patient 3]** 18A* polyps *MUTYH* c.303C>G (p.Tyr101X) homozygous

Male. Colonoscopy at 47 year of age following genetic diagnosis of MAP undertaken for family history of polyposis, identified >100 polyps with more marked proximal than distal colonic disease and relative sparing of rectum. Polyps for this study obtained at colectomy at 49 years of age. Otherwise well.

**[Patient 4]** 10A* polyps *MUTYH* c.536A>G (p.Tyr179Cys), c.1187G>A (p.Gly396Asp)

Male. Multiple polyps identified at colonoscopy at 14 years of age undertaken because of family history of polyposis. Colectomy at 16 years of age revealed > 100 polyps throughout colorectum. All polyps taken at colectomy.

**[Patient 10]** 1A polyps *MUTYH* c.536A>G (p.Tyr179Cys), c.1187G>A (p.Gly396Asp)

Male. Several adenomas removed at colonoscopy undertaken at 48 years of age because of early onset rectal cancer in a sibling. Further adenomas on follow up colonoscopy led to colectomy at 59 years revealing approximately 50 polyps. All polyps taken at colectomy.

1. **FAP Patients**

**[Patient 5]** 12A* polyps *APC* c.1999 C>T (p.Gln667X)

Female. Presented symptomatically with CRC and at least 69 polyps at 37 years. All polyps taken at colectomy at 37 years.

**[Patient 6]** 2J polyps *APC* c.477 C > G (p.Tyr159X)

Male. Genetic testing undertaken for family history of polyposis confirmed diagnosis of FAP at 39 years. Colonoscopy at 44 years confirmed dense polyposis. Colectomy at 45 years revealed approx. 600 polyps. All polyps taken at colectomy at 45 years.

**[Patient 7]** 5A* polyps *APC* exon 11 and 12 deletion

Female. Colonoscopy at 33 years for family history of polyposis. Colectomy at 35 years. Approx. 50 polyps identified. All polyps taken at colectomy at 35 years.

**[Patient 8]** 9A* polyps *APC* c.2805 C>A (p.Tyr935X)

Female. Several 100s of polyps at first colonoscopy undertaken for rectal bleeding at 21 yrs. Colectomy at 21 years. All polyps taken at 21 years of age.

**[Patient 9]** 15A polyps *APC* Exon 4 splice site c.423-1 G>A

Male. Genetic testing undertaken for family history of FAP. Colonoscopy showed multiple polyps. Colectomy at 27 years revealed approximately 70 polyps. All polyps taken at colectomy.

**[Patient 11]** 2G polyps *APC* c.477 C > G (Y159X)

Male. Genetic testing undertaken for family history of FAP leading to colonoscopy confirming several hundred small polyps and colectomy at 36 years. All polyps taken at colectomy.

**[Patient 12]** 3A polyps *APC* c.3366-3369 del TCAA

Male. Genetic testing undertaken for family history of FAP leading to colonoscopy confirming polyposis and colectomy at 19 years revealing 153 polyps. All polyps taken at colectomy.

**[Patient 13]** 11A polyps *APC* c.4393-4394 del AG

Male. Investigated at 35 years because of family and personal history of desmoids. Colonoscopy identified polyps throughout colorectum. FAP confirmed by genetic testing. Colectomy at 37 years revealed approximately 300 polyps. All polyps taken at colectomy.

**[Patient 14]** 17A polyps *APC* c.994C>T (p.Arg332X)

Male. Investigated at 44 years for abdominal pains and family history of colon cancer. Found to have over 100 polyps throughout colon, predominantly right sided. Colectomy at 44 years of age. All polyps taken at colectomy.
